# Morphological description and genetic analysis of a new black fly species (Diptera: Simuliidae) in the subgenus *Asiosimulium* from central Thailand

**DOI:** 10.1186/s13071-024-06441-z

**Published:** 2024-09-05

**Authors:** Wichai Srisuka, Hiroyuki Takaoka, Kritsana Taai, Wanchai Maleewong, Kittipat Aupalee, Atiporn Saeung

**Affiliations:** 1 Entomology Section, Queen Sirikit Botanic Garden, Chiang Mai, 50180 Thailand; 2 Tropical Infectious Diseases Research and Education Centre (TIDREC), Higher Institution Centre of Excellence (HICoE), Universiti Malaya, 50603 Kuala Lumpur, Malaysia; 3 Faculty of Veterinary Medicine, Western University, Kanchanaburi, 71170 Thailand; 4 Department of Parasitology, Faculty of Medicine, Khon Kaen University, Khon Kaen, 40002 Thailand; 5 Parasitology and Entomology Research Cluster (PERC), Department of Parasitology, Faculty of Medicine, Chiang Mai University, Chiang Mai, 50200 Thailand

**Keywords:** Aquatic Diptera, Biodiversity, *COI*, DNA barcode, Insect, Black flies, Simuliidae, *Simulium kittipati*

## Abstract

**Background:**

Black flies are among the most medically and veterinary important insects, as adult females of certain species are the sole vector of *Onchocerca volvulus*. Here, a new black fly species belonging to the subgenus *Asiosimulium* Takaoka & Choochote, 2005, is described and formally named as *Simulium* (*Asiosimulium*) *kittipati* sp. nov.

**Methods:**

Pupae and larvae of black flies were collected from available substrates in the stream from central Thailand. Pupae were individually separated in plastic tubes and maintained until adult flies emerged. The emerged adult flies associated with their pupal exuviae and cocoon as well as mature larvae preserved in 85% ethanol were used to describe the new species based on an integrated approach of morphological examination and molecular analysis of the *COI* gene.

**Results:**

The new species is characterized in the female by the medium-long sensory vesicle with a medium-sized opening apically, scutum with three faint longitudinal vittae, and the ellipsoidal spermatheca; in the male by the number of upper-eye (large) facets in 20 vertical columns and 21 horizontal rows, hind basitarsus slender, nearly parallel-sided, and median sclerite much wider and upturned apically; in the pupa by the head and thoracic integument densely covered with tiny tubercles, and the pupal gill of arborescent type with 28–30 filaments; and in the larva by the postgenal cleft deep, nearly reaching the posterior margin of the hypostoma, and dark pigmented sheath of the subesophageal ganglion. The DNA barcode successfully differentiated the new species from its congeners with an interspecific genetic divergence of 1.74–18.72%, confirming the morphological identification that the species is a new member of the subgenus *Asiosimulium*. Phylogenetic analyses also indicated that the new species is genetically closely related to *Simulium phurueaense* Tangkawanit, Wongpakam & Pramual, 2018, further supporting its morphological classification.

**Conclusions:**

This is the ninth species assigned to the subgenus *Asiosimulium* within the genus *Simulium* Latreille, 1802. Taxonomic notes and identification keys are given to distinguish this new species from the eight known species members in its same subgenus. Additionally, a distribution map of all species members in this subgenus occurring in Thailand and other countries is provided.

**Graphical abstract:**

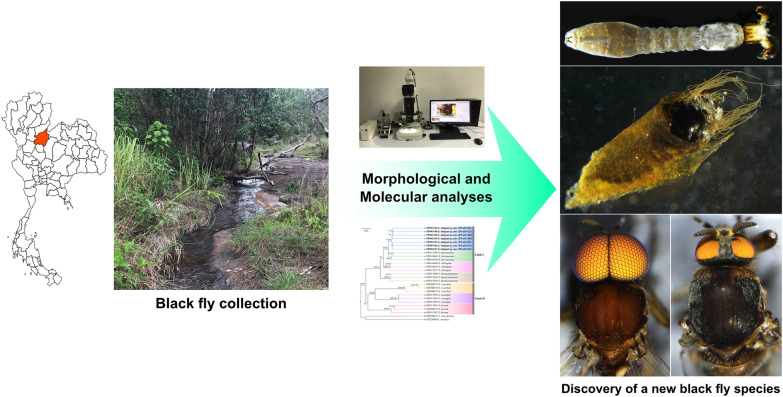

**Supplementary Information:**

The online version contains supplementary material available at 10.1186/s13071-024-06441-z.

## Background

Black flies (Diptera: Simuliidae) are small, robust dipterans (1–5 mm) notorious for their significant economic impact as biting and annoying pests of humans, domestic animals and livestock [1]. There are 2424 species (2407 living and 17 fossil) recorded worldwide [2].

*Simulium* Latreille, 1802 is the largest genus of Simuiidae, comprising more than 1900 member species in 43 subgenera. This genus has unique characteristics, such as wing without basal medial cell, hind basitarsus with calcipala, and tarsomere I of hind leg with pedisulcus [2, 3]. They are distributed globally and can be found in nearly all habitats (excluding Antarctica and some deserts and isolated oceanic islands), wherever suitable lotic microhabitats exist for their aquatic life stages (egg, larva, and pupa) [1, 2, 4]. Phylogenetic analysis of the large nuclear ribosomal subunit (28S), elongation factor-one alpha (EF-1α), and phosphoenolpyruvate carboxykinase (PEPCK) revealed that *Simulium* s.l. is the sister genus of *Metacnephia* Crosskey, 1969 [5].

The bite and subsequent blood-sucking by adult females can cause serious medical problems for humans and other vertebrates during daylight hours [1, 6]. Almost 98% of the world’s recorded black fly species feed on vertebrate (avian and mammalian) blood, and most of these species are likely to transmit blood-borne pathogens [7].

Certain species of black flies, such as *Simulium damnosum* Theobald, 1903 (complex) are the principal vectors of human onchocerciasis or river blindness, which is caused by the parasitic worm *Onchocerca volvulus* Leuckart, 1893 [8]. While over 99% of infected individuals live in 31 African countries, the disease is also found in certain areas of Latin America (the Yanomami Indigenous Land in Brazil and Venezuela) and Yemen [9]. In Japan, despite no cases of human onchocerciasis, several human cases of zoonotic onchocerciasis caused by *O*. *japonica* Yamaguti, 1934, a parasite of wild boar, and transmitted by *Simulium bidentatum* (Shiraki, 1935), have been reported [10–12]. Many man-biting species also cause allergic reactions in humans, sometimes leading to the clinically recognized syndrome of black fly fever [13]. Moreover, some species are transmitters of protozoan blood parasites of the genus *Leucocytozoon* Berestneff, 1904, which causes leucocytozoonosis, an often fatal disease affecting turkeys, ducks, geese and chickens. They can also transmit several species of filarial nematode worms of the genus *Onchocerca* to cattle, resulting in bovine onchocerciasis [1, 10].

Furthermore, black fly bites can lead to significant production losses in livestock, reducing both cattle milk yields and poultry egg production [1]. Apart from their negative impacts on humans and animals, black flies are considered valuable indicators to assess stream health in an urbanizing area [14]. Additionally, in northern Thailand, the larvae of *S*. *rudnicki* Takaoka & Davies, 1995, are collected as food by some hill tribes [15].

*Asiosimulium* Takaoka & Choochote, 2005, a small subgenus, is exclusively found in the Oriental region [16]. It is the second smallest subgenus in Thailand where a total of 145 valid species across six subgenera of the genus *Simulium* s.l. are formally documented: 6 species in *S.* (*Asiosimulium*) Takaoka & Choochote, 2005, 2 in *S*. (*Daviesellum*) Takaoka & Adler, 1997, 66 in *S*. (*Gomphostilbia*) Enderlein, 1921, 6 in *S*. (*Montisimulium*) Rubtsov, 1974, 10 in *S*. (*Nevermannia*) Enderlein, 1921 and 55 in *S*. (*Simulium*) [2, 17]. This subgenus is known to have distinct morphological characteristics, such as the cibarium armed with numerous spinous processes in the middle area and an elongated cercus in the female, the style shorter than the coxite and the paramere without hooks in the male, the gill of arborescent type (18–56 filaments) in the pupa, and the postgenal cleft deep in the larva [16, 18].

Few phylogenetic studies on this subgenus were conducted, but no clear relationships were established. For example, multi-gene phylogenetic analyses inferred from cytochrome c oxidase I, II, and 18S rRNA/ITS1 genes supported a sister group relationship between *Asiosimulium* and *Nevermamnia* [19], whereas sequence analysis based on the mitochondrial 16S ribosomal RNA (rRNA) gene indicated that *Asiosimulium* had no sister relationship with any of eight other subgenera analysed [20].

**Fig. 1:**
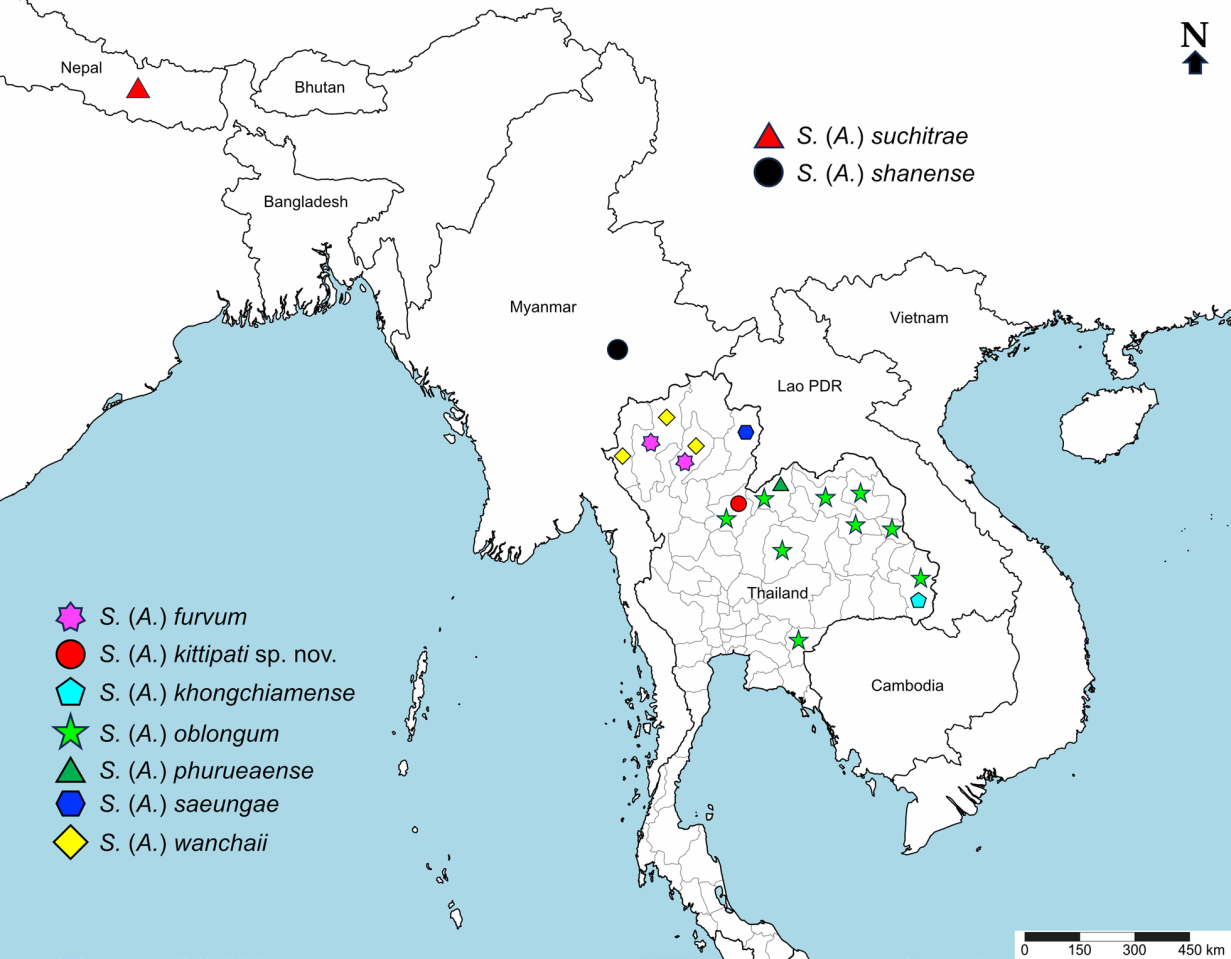
Map showing the distribution of all nine known species of black flies in the subgenus *Asiosimulium*

Among the eight described species in the subgenus *Asiosimulium*, six have been reported from Thailand: *Simulium* (*Asiosimulium*) *furvum* Takaoka & Srisuka, 2013, *S*. (*A*.) *oblongum* Takaoka & Choochote, 2005, *S*. (*A*.) *phurueaense*, *S*. (*A*.) *saeungae* Takaoka & Srisuka, 2018, *S*. (*A*.) *wanchaii* Takaoka & Choochote, 2006 and *S*. (*A*.) *khongchiamense* Takaoka, Srisuka & Saeung, 2023 [17]. Meanwhile, the remaining two species, *S*. (*A*.) *shanense* Takaoka, Srisuka & Saeung, 2017 [21] and *S*. (*A*.) *suchitrae* Takaoka, 2010 [22], occur in Myanmar and Nepal, respectively (Fig. 1).

Mostly, the pupae, and larvae of all Thai species members are found in temporary streams with slow water flow rates during the rainy season on rock surfaces, roots, and grass trailing in water, exposed to sunlight at different elevations (127–1420 m) [17, 23]. The medical and veterinary significance of all species members in this subgenus remains largely undetermined. However, using a human attractant, two adult females of *S.* (*A*.) *wanchaii* were caught in Doi Suthep-Pui National Park, Chiang Mai province [24], and several adult females of this species were also collected at Phu Langka, Phayao province, northern Thailand (unpublished data).

Recently, one undescribed species belonging to the subgenus *Asiosimulium* was collected from Nakhon Thai district, Phitsanulok province, central Thailand. On the basis of the morphological and molecular evidence, it is described as a new species. Furthermore, to aid in species identification, the updated identification keys to species as well as the morphological characteristics that distinguish this new species from other species in the same subgenus are provided.

## Methods

### Collection and preparation of specimens

Pupae and larvae of black flies were manually collected from available substrates including trailing grasses, fallen leaves, rock surfaces and tree roots in the stream. All larvae collected from the field were sorted in laboratory and only mature larvae were preserved individually in 85% ethanol. Individual pupae were maintained in a plastic tube (15 ml) with moist filter paper until adults emerged. After emergence, adult flies were kept alive in the same tube for at least 24 h to secure hardening and colouring of their body and legs. Then, each adult fly, associated with its pupal exuviae and cocoon, was preserved in 85% ethanol [6].

### Morphological analysis

Overall 20 adults (10 females and 10 males reared from pupae, as well as their associated pupal exuviae and cocoons), and 10 mature larvae (Table 1), were used for morphological analysis. Description methods, as well as the morphological terminology, followed those outlined by Takaoka [25] and partially by Adler et al. [4]. The photography method adhered to the protocol described previously by Srisuka et al. [26]. Morphological comparisons with all other known species of the subgenus *Asiosimulium* were performed using the original descriptions by Takaoka and Shrestha [22], Takaoka and Choochote [24], Takaoka et al. [16, 21, 27], Srisuka et al. [23], Tangkawanit et al. [28], and Aupalee et al. [17]. In addition, the new species was morphologically compared with the type specimens of all species members of this subgenus (except for *S*. *suchitrae* and *S*. *phurueaense*), deposited in the Entomology Section of the Queen Sirikit Botanic Garden (QSBG), Chiang Mai, Thailand.

**Table 1:** Data on the types of specimens of *S*. *kittipati* sp. nov. deposited in the QSBG, Chiang Mai, Thailand

Species (stage)	Collection code	No	Collection site	GPS coordinate	Altitude (m)	Collection date	Status	Preservation
*S*. *kittipati* sp. nov. (female)	QSBG2023–49–1	1	Nakhon Thai, Phitsanulok	17° 00′ 27.6ʺ N 100° 59′ 21.0ʺ E	1123	15 July 2023	Holotype	85% ethanol (associated pupal exuviae and cocoon)
*S*. *kittipati* sp. nov. (female)	QSBG2023–49–2 to–10	9	Nakhon Thai, Phitsanulok	17° 00′ 27.6ʺ N 100° 59′ 21.0ʺ E	1123	15 July 2023	Paratype	85% ethanol (associated pupal exuviae and cocoon)
*S*. *kittipati* sp. nov. (male)	QSBG2023–49–11 to –20	10	Nakhon Thai, Phitsanulok	17° 00′ 27.6ʺ N 100° 59′ 21.0ʺ E	1123	15 July 2023	Paratype	85% ethanol (associated pupal exuviae and cocoon)
*S*. *kittipati* sp. nov. (mature larva)	QSBG2023–49–21 to –30	10	Nakhon Thai, Phitsanulok	17° 00′ 27.6ʺ N 100° 59′ 21.0ʺ E	1123	15 July 2023	Paratype	85% ethanol

### Genetic analysis

Seven specimens of *Simulium kittipati* sp. nov. including two females (PSAS1-F1, PSAS1-F2), two males (PSAS1-M1, PSAS1-M2) and three larvae (PSAS1-L1, PSAS1-L2, PSAS1-L3) were selected for molecular analysis (Table 2). Genomic DNA extraction from either the thorax of adult flies or abdominal segments 1–5 of larvae was performed using TIANamp Genomic DNA Kit (TIANGEN Biotech, Beijing, China), following the manufacturer’s instructions.

**Table 2:** Details of the *COI* sequences of *Simulium kittipati* sp. nov. and its other six known related species of the subgenus *Asiosimulium* used for molecular analysis in this study

Species (no.)	Stage (no.)	GenBank accession number	Collection site	GPS coordinates	Altitude (m)	References
*S*. *kittipati* sp. nov. (7)	F (3)	PP693144, PP693145	Nakhon Thai, Phitsanulok	17° 00′ 27.6ʺ N 100° 59′ 21.0ʺ E	1123	This study
	M (2)	PP693149, PP693150				
	L (3)	PP693146–PP693148				
*S. khongchiamense* (3)	F (1)	OR115594	Khong Chiam, Ubon Ratchathani	15° 24′ 16ʺ N 105° 30′ 55ʺ E	220	[17]
	M (1)	OR115600				
	L (1)	OR115596				
*S*. *oblongum* (3)	L (1)	OR115593	Phuphan, Sakon Nakhon	17° 07′ 24ʺ N 104° 01′ 07ʺ E	344	[17]
	N/A (2)	MF616447, MF616448	Phupakud 2, Mukdahan	N/A	N/A	[28]
*S*. *phurueaense* (3)	L (1)	OR115580	Phu Ruea, Loei	17° 29′ 59ʺ N 101° 20′ 09ʺ E	1160	[17]
	N/A (2)	MF616459, MF616461	Phu Ruea, Loei	17° 30′ 20ʺ N 101° 20′ 30ʺ E	1220	[28]
*S*. *wanchaii* (3)	N/A (3)	MZ508533–MZ508535	Hot, Chiang Mai/Khun Yuam, Mae Hong Son	18° 09′ 23ʺ N 98° 23′ 18ʺE/18° 53′ 24ʺ N 98° 35′ 18ʺ E	1100/1380	[29]
*S*. *furvum* (3)	L (2)	OR115581, OR115582	Thoen, Lampang	17° 37′ 19ʺ N 99° 18′ 49ʺ E	468	[17]
	N/A (1)	MZ508532	Hot, Chiang Mai	18° 09′ 23ʺ N 98° 23′ 18ʺ E	1100	[29]
*S*. *saeungae* (3)	M (1)	OR115589	Khun Sathan, Nan	18° 16′ 44ʺ N 100° 30′ 14ʺ E	1316	[17]
	L (2)	OR115585, OR115586				

*F* Female, *M* Male, *L* Larva, *N/A* Not available

The mitochondrial *COI* gene (685 bp) was amplified using the LCO1490 and HCO2198 primer pair [30]. The final volume of each PCR reaction was 20 μl, consisting of 2 µl of DNA template, 1 U of *Taq* DNA polymerase, 3 mM of MgCl_2_, 0.25 mM of deoxynucleotide triphosphate (dNTP) and 0.2 µM of each primer. The optimal PCR protocol comprised an initial denaturation at 94 °C for 2 min followed by 40 cycles of denaturation at 94 °C for 30 s, annealing at 50 °C for 45 s and extension at 72 °C for 45 s, with a final 5 min extension step at 72 °C. To assess the success of the amplification, PCR products were run on a 1.5% agarose gel with the 100 bp DNA marker, and stained with Ultrapower™ (BioTeke, Beijing, China) dye. The unpurified PCR products were subsequently sent to First Base Laboratories Sdn Bhd (Malaysia) for purifying and sequencing using the BigDye®Terminator v.3.1 cycle sequencing kit on an ABI 3730XL Genetic Analyzer (Applied Biosystems Inc., Foster City, CA, USA).

To generate the consensus sequence, both forward and reverse sequences of each sample were assembled, aligned and edited manually using Geneious Prime 2024.0.4 [31]. Intraspecific and interspecific divergence values were calculated on the basis of the Kimura 2-parameter (K2P) model, executed in MEGA 11 [32, 33]. The evolutionary relationships between the new species and other related species of the subgenus *Asiosimulium* were inferred using neighbour-joining (NJ) and maximum likelihood (ML) methods. The NJ tree was built under the K2P model using MEGA 11 with 1000 bootstrap replicates, while the ML tree was constructed using IQ-TREE version 2.3.1 [34] with 10,000 ultrafast bootstrap replicates [35]. The best-fit evolutionary model for the ML method (HKY + F + G4) was determined using ModelFinder implemented in IQ-TREE based on the Bayesian Information Criterion (BIC) [36].

Except for *Simulium shanense* and *S*. *suchitrae* which lack their *COI* sequences in the GenBank database, six other known species of the subgenus *Asiosimulium*, namely *S*. *furvum*, *S*. *khongchiamense*, *S*. *oblongum*, *S*. *phurueaense*, *S*. *saeungae* and *S*. *wanchaii* were included in the phylogenetic analyses (Table 2). The *COI* sequences of *S.* (*Nevermannia*) *aureohirtum* Brunetti, 1911 and *Parasimulium crosskeyi* Peterson, 1977 were selected as the outgroup species. All new sequences generated have been deposited in the GenBank database and are available under accession numbers: PP693144–PP693150.

## Results

### Description of a new species

Family Simuliidae Newman, 1834.

Genus *Simulium* Latreille, 1802.

Subgenus (*Asiosimulium*) Takaoka & Choochote, 2005.

*Simulium* (*Asiosimulium*) *kittipati* Srisuka, Takaoka & Saeung sp. nov.

**Fig. 2:**
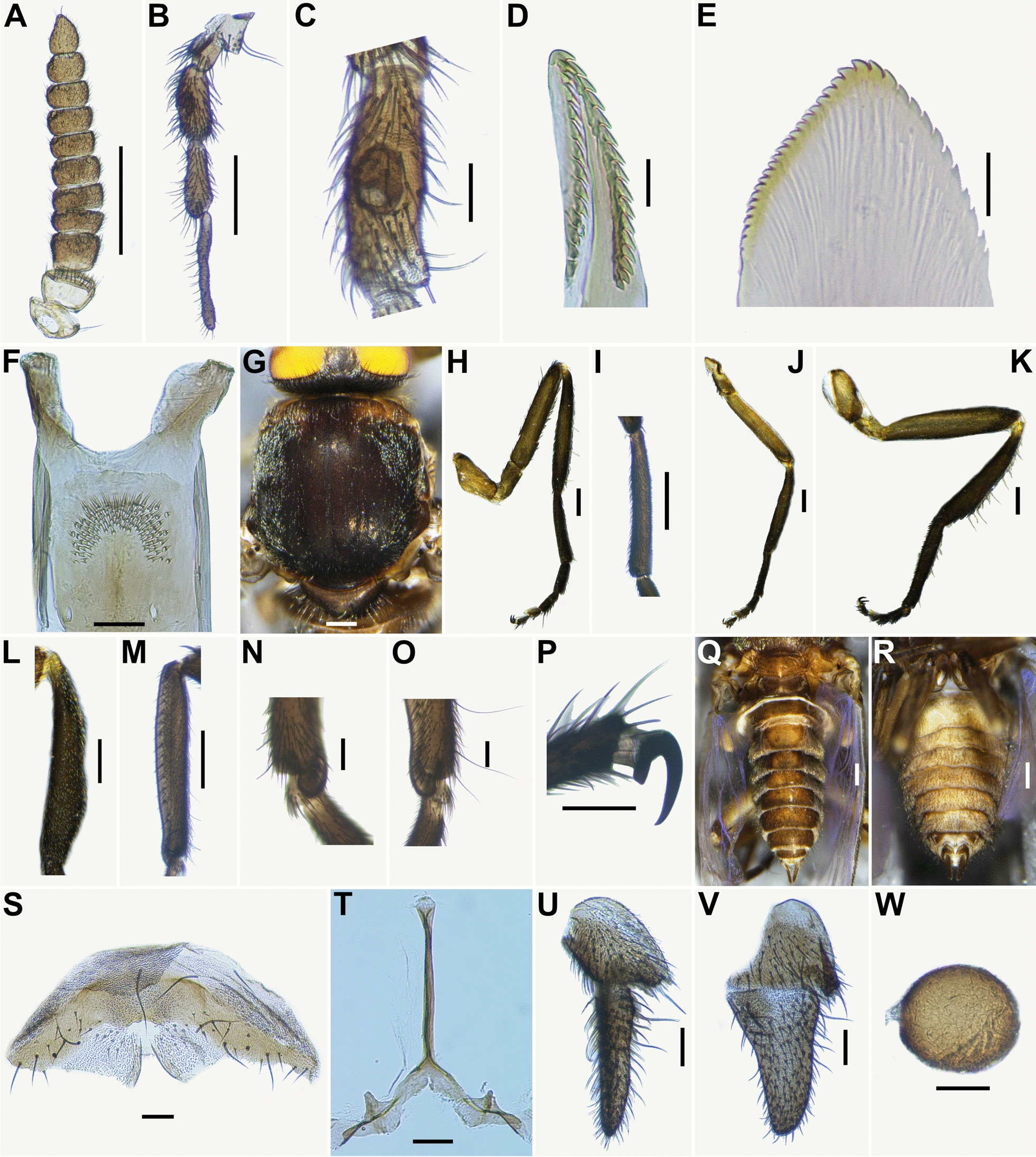
Female of *Simulium kittipati* sp. nov. **A** antenna, **B** maxillary palpus, **C** sensory vesicle (left side; front view), **D** lacinia, **E** mandible, **F** cibarium (front view), **G** thorax (dorsal view), **H** foreleg (left side; outer view), **I** fore basitarsus (left side; outer view), **J** midleg (left side; outer view), **K** hind leg (left side; outer view), **L** hind tibia (left side; outer view), **M** hind basitarsus and second tarsomere, **N** calcipala (left side; inner view), **O** pedisulcus, **P** claw (lateral view), **Q** and **R** abdomens (**Q** dorsal view; **R** ventral view), **S** eighth sternite and ovipositor valves (ventral view), **T** genital fork (ventral view), **U** and **V** paraprocts and cerci (**U** ventral view; **V** lateral view), **W** spermatheca (lateral view). Scale bars. 0.2 mm for **A**, **B**, **G**–**M**, **Q** and **R**; 0.05 mm for **C**, **F**, **N**–**P** and **S**–**W**; 0.02 mm for **D** and **E**

**Fig. 3:**
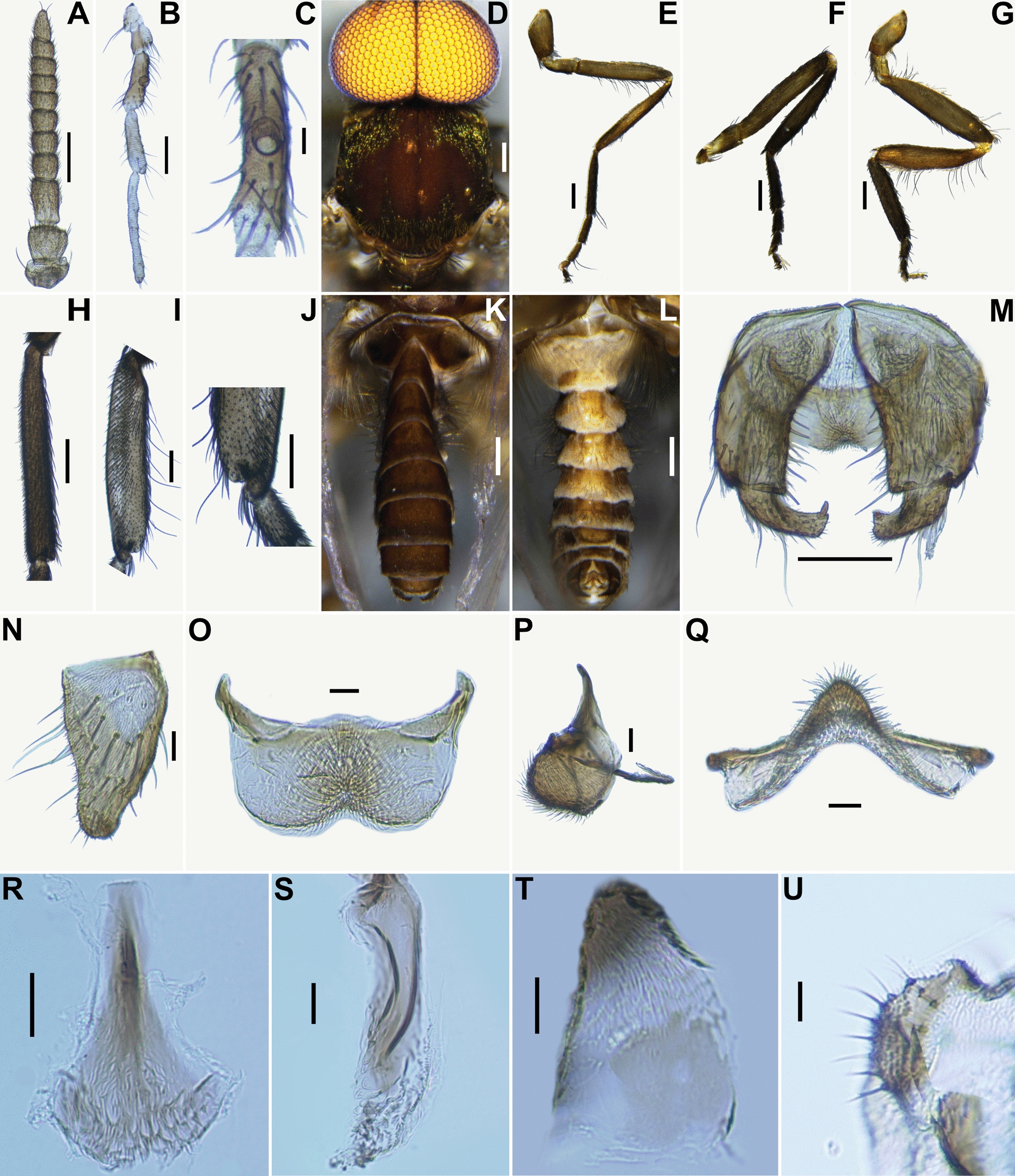
Male of *Simulium kittipati* sp. nov. **A** antenna, **B** maxillary palpus, **C** sensory vesicle (left side; front view), **D** thorax (dorsal view), **E** foreleg (left side; outer view), **F** midleg (left side; outer view), **G** hind leg (left side; outer view), **H** fore basitarsus (left side; outer view), **I** hind basitarsus and second tarsomere (left side, outer view), **J** calcipala and pedisulcus (left side, inner view), **K** and **L** abdomens (**K** dorsal view; **L** ventral view), **M** coxites, styles and ventral plate (ventral view), **N** style (ventrolateral view), **O**–**Q** ventral plate (**O** ventral view; **P** lateral view; **Q** caudal view), **R** median sclerite (ventral view), **S** paramere, **T** aedeagal membrane and dorsal plate, **U** cerci (lateral view). Scale bars. 0.2 mm for **D**–**G**, **K** and **L**; 0.1 mm for **A**, **B**, **H**–**J** and **M**; 0.02 mm for **C**, **N**–**U**

**Fig. 4:**
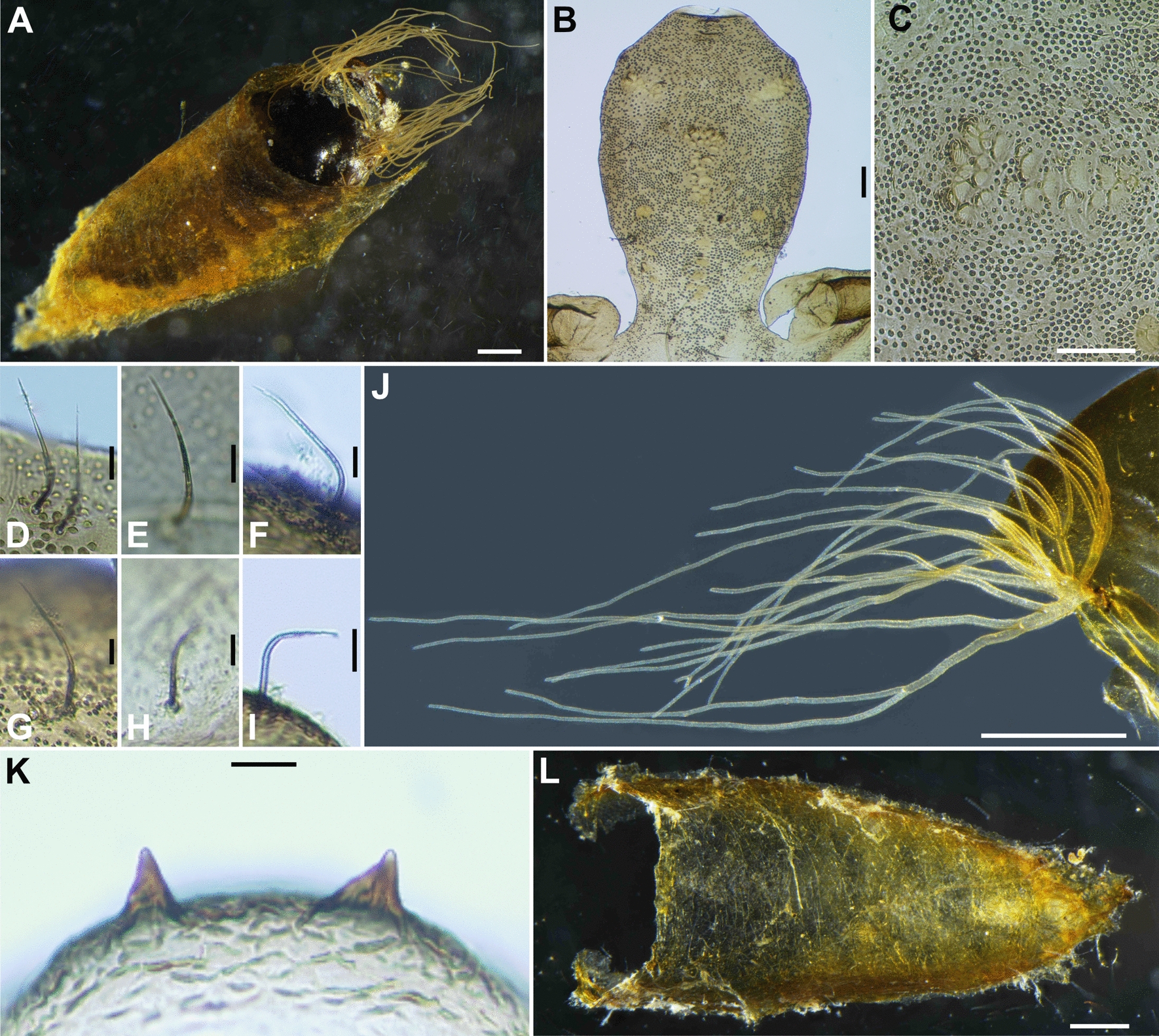
Pupa of *Simulium kittipati* sp. nov. **A** pupa with its cocoon (dorsolateral view), **B** frons, **C** tubercles on frons, **D** frontal trichome, **E** facial trichome, **F**–**I** thoracic trichomes (**F** anterodorsal; **G** anterolateral; **H** mediolateral; **I** ventrolateral), **J** gill filaments (left side; lateral view), **K** terminal hooks (caudal view), **L** cocoon (dorsal view). Scale bar. 0.5 mm for **A**, **J** and **L**; 0.1 mm for **B** and **C**; 0.02 mm for **D**–**I** and **K**

**Fig. 5:**
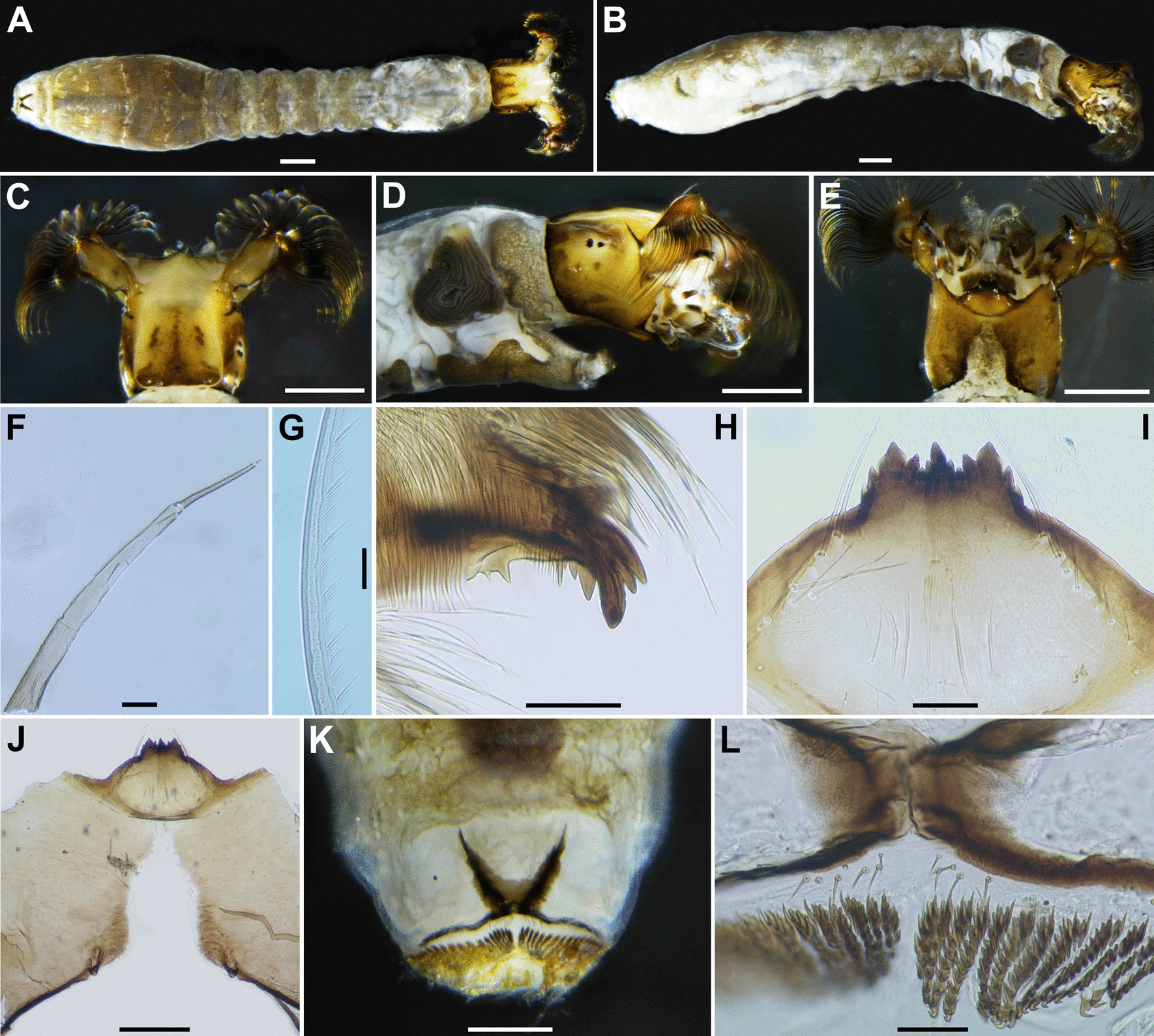
Larva of *Simulium kittipati* sp. nov. **A** and **B** whole body (**A** dorsal view; **B** lateral view), **C**–**E** head capsule (**C** dorsal view; **D** lateral view; **E** ventral view), **F** antenna, **G** microtrichia, **H** apical portion of mandible, **I** hypostoma, **J** postgenal cleft (ventral view), **K** anal sclerite and posterior circlet (dorsal view), **L** sensilla. Scale bars. 0.5 mm for **A**–**E**; 0.2 mm for **J** and **K**; 0.05 mm for **F**,** H, I** and **L**; 0.02 mm for** G**

**Diagnosis** *Simulium* (*Asiosimulium*) *kittipati* sp. nov. can be distinguished from other species of the subgenus *Asiosimulium* by the combination of the following characteristics: female: sensory vesicle 0.25–0.35 times as long as third palpal segment (Fig. 2C). Scutum with three faint longitudinal vittae (Fig. 2G). Spermatheca ellipsoidal (Fig. 2W). Male: upper-eye (large) facets in 20 vertical columns and 21 horizontal rows. Ventral plate wide, with posterior margin moderately concave medially when viewed ventrally (Fig. 3O). Medial sclerite plate-like, widened and rounded with several sutures and upturned apical (Fig. 3R). Pupa: Gill arborescent, with 28–30 short to medium-long slender thread-like filaments (Fig. 4J). Larva: postgenal cleft deep, nearly reaching posterior margin of hypostoma (Fig. 5E and J). Sheath of subesophageal ganglion dark pigmented (Fig. 5E).

### Description

Female (*n* = 10): Body length 3.2–3.5 mm (mean 3.2 mm).

*Head*: slightly narrower than thorax. Frons brownish black, densely covered with whitish-yellow hairs interspersed with several dark brown longer and stouter hairs along each lateral margin; frontal ratio 1.42–1.53:1.00:1.74–1.78. Frons-head ratio 1.00:3.32–4.26. Fronto-ocular area well developed, triangular, directed outward and slightly upward. Clypeus medium brown, densely covered with whitish-yellow hairs intermixed with dark brown longer and stouter hairs. Labrum 0.84–0.90 times as long as clypeus. Antenna (Fig. 2A) composed of scape, pedicel and nine flagellomeres, medium to dark brown, except scape, pedicel and base of first flagellomere yellow, first flagellomere 1.2–1.6 times as long as second. Maxillary palpus (Fig. 2B) consisting of five segments, grayish to medium brown except third segment dark brown and segment four medium brown on outer surface and grayish on inner surface, proportional lengths of third, fourth, and fifth segments 1.00:0.95–1.18:0.67–0.88; third segment moderately swollen; sensory vesicle (Fig. 2C) ellipsoidal, 0.30 times as long as third segment, with medium-sized opening apically (Fig. 2C). Lacinia (Fig. 2D) with 15 or 16 inner and 13 outer teeth. Mandible (Fig. 2E) with 21 inner and 11 or 12 outer teeth. Cibarium (Fig. 2F) moderately concave posterodorsally and with 116–119 spinous processes medially.

*Thorax* (Fig. 2G): scutum brownish black to black (Fig. 2G) except anteromedian portion widely medium brown and anterolateral calli light brown, shiny and white pruinose when illuminated at certain angles and scutum with three faintly indistinct dark-brown narrow longitudinal vittae (one median, two submedian) (Fig. 2G) when illuminated in front and viewed dorsally, only median longitudinal vittae observed when illuminated posteriorly and viewed dorsally, scutum densely covered with yellowish-white short hairs interspersed with several dark-brown upright long hairs on prescutellar area, Scutellum medium to dark brown, moderate covered with yellowish-white short hairs interspersed with dark-brown upright long hairs on lateral margin. Postnotum medium to dark brown, white pruinose when illuminated at certain angles and bare. Pleural membrane bare. Katepisternum longer than deep, dark brown to brownish black, thinly white pruinose and bare.

*Legs*: foreleg (Fig. 2H): coxa light brown; trochanter medium brown; femur light brown with apical cap medium brown; tibia dark brown except extreme base yellow and median portion widely light brown; tarsus dark brown to brownish black, with moderate dorsal hair crest; basitarsus (Fig. 2I) nearly parallel-sided, 6.0–6.5 times as long as its greatest width. Midleg (Fig. 2J): coxa dark brown except posterolateral surface brownish black; trochanter light brown; femur light brown with apical cap medium brown; tibia dark brown except extreme base yellow and median portion of outer and inner surfaces widely light brown; tarsus dark brown to brownish black. Hind leg (Fig. 2K): coxa medium brown; trochanter light brown; femur light brown with apical cap dark brown; tibia (Fig. 2L) dark brown except extreme base yellow and median portion of outer and inner surface widely median brown; tarsus medium brown except basal half of second tarsomere light brown; basitarsus (Fig. 2M) nearly parallel-sided on basal two-thirds, then somewhat narrowed towards apex, 6.0–6.6 times as long as wide and 0.63–0.71 and 0.52–0.63 times as wide as greatest widths of tibia and femur, respectively; calcipala (Fig. 2N) well developed, 1.2–1.5 times as long as its width at base, and 0.33–0.38 times as wide as greatest width of basitarsus. Pedisulcus (Fig. 2O) well defined. Claw (Fig. 2P) with long basal tooth 0.40 times length of claw.

*Wing*: length 2.7–3.1 mm, mean 3.0 mm (*n* = 20). Costa with dark short spinules and dark brown hairs. Subcosta with dark hairs except near apex bare. Hair tuft on stem vein dark brown. Basal portion of radius fully haired. R_1_ with dark spinules and hairs. R_2_ with dark hairs only. Basal cell and basal median cell absent.

*Halter*: with pale stem and medium brown knob.

*Abdomen*: basal scale light brown, with fringe of yellowish-white long hairs. Dorsal and lateral surfaces of abdomen medium brown to brownish black, densely covered with yellowish-white recumbent short hairs interspersed with dark brown long hairs; dorsum of segments two to seven (Fig. 2Q) slightly shiny when illuminated at certain angles; ventral surface of abdomen (Fig. 2R) medium brown except segments two to four greyish white to light brown, moderately covered with dark brown setae except segments two and three sparsely covered with dark brown setae; segment seven without sternal plate.

*Terminalia*: sternite eight (Fig. 2S) wide, bare medially but furnished with eight to ten medium-long and five or six long dark hairs on each side. Ovipositor valve (Fig. 2S) nearly triangular, thin, membranous except inner margin narrowly sclerotized, densely covered with microsetae interspersed with seven to ten short hairs; inner margins, straight or slightly convex, moderately separated from each other. Genital fork (Fig. 2T) inverted-Y-shaped, with well sclerotized stem, relatively wide, well sclerotized arms each having distinct long projection directed anterodorsally, and deep incision between arms. Paraproct in ventral view (Fig. 2U) subquadrate, with distinct process produced ventrally along anteromedial margin, with 10–12 colourless sensilla on darkened anteromedial surface; paraproct in lateral view (Fig. 2V) about 0.8 times as long as wide, slightly produced ventrally beyond ventral margin of cercus, and with numerous short to medium-long hairs on lateral and ventral surfaces. Cercus in ventral view (Fig. 2U) slightly parallel-sided, gradually narrowed posteriorly; cercus in lateral view (Fig. 2V) gradually narrowed posteriorly, with rounded apex, 1.6 times as long as its width at base. Spermatheca (Fig. 2W) ellipsoidal, 1.2 times as long as its width, strongly sclerotized except base of duct widely unsclerotized, with distinct reticulate surface pattern; internal setae not discernible.

Male (*n* = 10): body length 3.1–3.5 mm (mean 3.3 mm).

*Head*: much wider than thorax. Holoptic. Upper eye medium brown, consisting of large facets in 20 vertical columns and 21 horizontal rows. Clypeus brownish black, thinly white pruinose, moderately covered with dark brown long hairs interspersed with yellow fine hairs along lateral margins. Antenna (Fig. 3A) composed of scape, pedicel and nine flagellomeres, medium to dark brown except scape pedicel and base of first flagellomere whitish yellow; first flagellomere somewhat elongate, 1.7–1.9 times as long as second. Maxillary palpus (Fig. 3B) composed of five segments, greyish brown except third segment dark brown; proportional lengths of third, fourth and fifth segments 1.00:1.06–1.1:1.38–1.53; third segment of moderate size; sensory vesicle (Fig. 3C) ellipsoidal, 0.25–0.28 times as long as third segment, with small opening apically.

*Thorax* (Fig. 3D): scutum dark brown (appears slightly different from female), except shoulders, wide portion along each lateral margin, scutum with three longitudinal vittae, one median and two submedian; more clearly than in females) when illuminated in front and viewed dorsally; two submedian vittae wider and darker than the median one when illuminated posteriorly and viewed dorsally and prescutellar area dark brown, shiny and white pruinose when illuminated at certain angles and moderately covered with yellowish-white short hairs interspersed with several dark-brown upright long hairs on prescutellar area. Scutellum medium brown, moderately covered with dark-brown upright long hairs interspersed with yellowish-white medium long hairs. Postnotum medium to dark brown, white pruinose when illuminated at certain angles and bare. Pleural membrane bare. Katepisternum dark brown and bare.

*Legs* (Fig. 3E–G): colour similar to female, though somewhat darker. Fore basitarsus (Fig. 3H) nearly parallel-sided, 8.1 times as long as its greatest width. Hind basitarsus (Fig. 3I) nearly parallel-sided, 4.70–5.30 times as long as its greatest width, 0.71–0.76 and 0.60–0.68 times as wide as greatest widths of hind tibia and femur, respectively; calcipala (Fig. 3J) well developed, 1.25 times as long as width at base and 0.58 times as wide as greatest width of basitarsus; pedisulcus (Fig. 3J) well developed.

*Wing*: length 2.3–3.0 mm, mean 2.7 mm (*n* = 20); other characteristics same as in female except subcosta bare.

*Abdomen*: sasal scale brownish black, with fringe of light-brown long hairs. Dorsal surface of abdomen (Fig. 3K) similar to female except narrow area along posterior margin of segments three to eight paler, moderately covered with light to medium brown hairs; second and fifth to eighth tergites each with pair of dorsolateral or lateral shiny patches when illuminated at certain angles, though tergites three and four each faintly with pair of shiny patches, too in one male; lateral surface of abdominal segments three and four moderately covered with medium–brown long hair (Fig. 3K); ventral surface (Fig. 3L) of abdominal segments two to five greyish white to light brown, and those of other segments medium brown; sternites two to six medium brown and somewhat shiny when illuminated ventrally and covered with medium brown hairs.

*Genitalia*: coxite in ventral view (Fig. 3M) rectangular, much longer than wide, 1.6 times as long as its greatest width. Style in ventral view (Fig. 3M) short, 0.51 times as long as coxite, gradually tapered towards apex, bent inward, rounded apically and with prominent apical spine; style in ventrolateral view (Fig. 3N) wide basally, 0.54 times as wide as long, tapered towards apex. Ventral plate in ventral view (Fig. 3O): body wide, 0.44 times as long as wide, with its posterior margin moderately concave medially, and densely covered with setae, except both anterolateral portions bare, and basal arms short, divergent anterolaterally; ventral plate in lateral view (Fig. 3P) moderately produced ventrally; ventral plate in caudal view (Fig. 3Q) inverted-V shaped and covered with setae on posterior surface. Median sclerite in lateral view (Fig. 3P) thin, arising near anterior margin of ventral plate directed dorsally and upturned apically; median sclerite in ventral view (Fig. 3R) plate-like, gradually widened from base to apex, with apical portion rounded and having several sutures. Paramere (Fig. 3S) well sclerotized, apical part with many lobes, no hook. Aedeagal membrane (Fig. 3T) densely covered with minute setae and relatively larger setae on lateral margins; dorsal plate (Fig. 3T) square in shape, lightly pigmented. Ventral surface of tenth abdominal segment without distinct hairs laterally near cercus on each side. Cercus (Fig. 3U) in form of narrow lobe, covered with 11–12 hairs.

Pupa (*n* = 10) (Fig. 4A): body length 3.8–4.8 mm (mean 4.4 mm).

*Head*: integument (Fig. 4B) yellowish to dark brown, densely covered with tiny round tubercles (Fig. 4C); frons with two unbranched medium-long trichomes (Fig. 4D) on each side; face with one short unbranched trichome (Fig. 4E) on each side, which is shorter than frontal trichomes; antennal sheath without any projection and tubercle.

*Thorax*: integument yellowish to dark brown, densely covered with tiny round tubercles; thorax on each side with two long trichomes anterodorsally (Fig. 4F), one long and two medium-long trichomes anterolaterally (Fig. 4G), one medium-long trichome mediolaterally (Fig. 4H), and three medium-long trichomes ventrolaterally (Fig. 4I); all trichomes unbranched. Gill (Fig. 4J) of arborescent type, composed of 28–30 short to medium-long slender thread-like filaments arranged in five or six groups; 28 filaments arranged as [(1 + 2) + (2 + 2)] + [2 + (2 + 2)] + [2 + 1 + 1 + 2)] + [1 + 2] + [2 + (2 + 2)] or [2 + (1 + 2)] + [2 + 1 + (2 + 1)] + 2 + [2 + 1 + 1 + 2] + [1 + 2] + [2 + (2 + 2)], or 29 filaments arranged as [(2 + 2) + (1 + 2)] + [2 + (2 + 2)] + [2 + 2 + (1 + 2)] + [1 + 2] + [2 + (2 + 2)] or [2 + (1 + 2)] + 2 + [2 + (2 + 2)] + [1 + 1 + 1 + 2 + 2] + [1 + 2] + [2 + (2 + 2)], or 30 filaments arranged as [2 + (2 + 2)] + [2 + 1 + (1 + 2)] + 2 + [(1 + 2) + (1 + 1 + 2)] + [1 + 2] + [2 + 2 + 2] or [(2 + 2) + (1 + 2)] + [2 + (2 + 2)] + [(1 + 2) + 1 + (2 + 2)] + [1 + 2] + [2 + 2 + 2] arising from short common basal stalk, which has moderately developed transparent basal fenestra at base; common basal stalk 0.28 times length of interspiracular trunk; all filaments light to medium brown, with longest filament 2.33–2.43 mm, with annular ridges and furrows, and covered densely with minute tubercles.

*Abdomen*: dorsally, all segments moderately sclerotized; segments one and two medium brown and moderately covered with tiny round tubercles; segment one with one medium-long slender seta on each side; segment two with one medium-long seta on lateral margin and a row of five short spinous setae on each side; segments three to four light brown, partially and weakly tuberculate, each with four stout hooks and two short spinous seta; segments five light brown with two medium long setae and three short setae on each side, without comb-like groups of minute spines and without spine-combs, segments six to nine light brown, each with comb-like groups of minute spines and without spine-combs on each side; segments six to seven with two medium long setae on each side; segment eight with two short setae on each side; segment nine with pair of small cone-shaped terminal hooks (Fig. 4K) and without small tubercles. Laterally, segment nine with two grapnel-shaped hooklets on each side. Ventrally, all segments transparent except segment nine yellowish; segment three with two medium long setae on each side; segment four with four medium long setae on each side and sparsely with comb-like groups of minute spines; segments five to seven each with pair of unbranched stout hooks on each side. Segments five with five medium long setae on each side; segments six to eight each with comb-like groups of minute spines on each side; segments six to seven each with three medium long setae on each side; segments eight with two short setae on each side.

*Cocoon* (Fig. 4L): yellow, wall-pocket shaped (slipper-shaped), thinly woven, individual threads visible or invisible; 3.8–4.8 mm long by 1.7–2.0 mm wide.

Larva (*n* = 10) (Fig. 5A, B): body length 7.6–8.1 mm (mean 7.8 mm). Body light to dark grey, thoracic first segment encircled with medium grey transverse band though disconnected ventromedially, metathoracic with narrow grey transvers band dorsally; abdominal segments one and two each medium to dark grey entirely and abdominal segments three to nine dark grey on dorsal and dorsolateral surface, and white on ventrolateral and ventral surface.

*Head*: cephalic apotome (Fig. 5C) length 0.91–1.07 mm (mean 9.8 mm), width 0.50–0.58 mm (mean 0.52 mm). Whitish yellow on anterior portion and deep yellow posterior except wide area along posterior margin dark brown and covered with colorless setae; head spots distinct, though posterolateral spots merged with darkened area and posterior spot of mediolongitudinal spots connected posteriorly to darkened area along posterior margin, anterior of mediolongitudinal spots with four light brown spots, anterolateral spot with two large dark brown spots. Lateral surface (Fig. 5D) of head capsule yellow except eye-spot region whitish and area above and posterior to eye-spot region dark brown while light brown on anterior region, with distinct dark brown spots, i.e., three isolated small spots below eye-spot region (all spot medium brown) and two large spots (dark brown) merged with darkened area along posterior margin. Ventral surface of head capsule (Fig. 5E) yellow to light brown except portions along both sides of postgenal cleft dark brown; elongate spots on each side of postgenal cleft indistinct, sparsely covered with minute colorless setae. Cervical sclerites each composed of anterior light brown elongate piece fused to occiput and posterior elliptical medium brown piece. Antenna (Fig. 5F): length 0.48–0.60 mm (mean 0.53 mm), consisting of three articles and apical sensillum, little longer than stem of labral fan (1.2 time as long as stem of labral fan); proportional lengths of first, second and third segments 1.0:0.83–0.86:0.70–0.77. Labral fan stem long 0.41–0.50 mm (mean 0.45 mm) with 41–42 main rays, each with one row of microtrichia (Fig. 5G) on posterior margin of main fan ray, consisting of 21 to 32 intervals of long microtrichia, each interval with 14 to 19 short microtrichia subequal length. Mandible (Fig. 5H) with mandibular serration consisting of 1 large tooth and 1 small tooth; large tooth at right angle to mandible on apical side; comb-teeth composed of three teeth, of which first is much longer and thicker than second and third; second is slightly longer than third; supernumerary serration absent. Hypostoma (Fig. 5I) with nine apical teeth in row; median tooth equal in length to corner teeth; lateral margin weakly serrated anteriorly; eight or nine hypostomal bristles per side. Postgenal cleft (Fig. 5E and J) deep, nearly reaching posterior margin of hypostoma 8.8 times as long as postgenal bridge, sheath of subesophageal ganglion dark pigmented.

*Thorax* and *Abdomen*: Thoracic and abdominal cuticles almost bare except both sides of anal sclerite and ventral bulge moderately covered with simple colorless setae, ventral surface of thoracic segments two and three each with dark gray plate. Rectal scales not discernible. Rectal organ compound, each of three lobes with 13–15 finger-like secondary lobules. Anal sclerite (Fig. 5K) X-shaped, anterior arms as long as posterior arms; anterior arms broadened, and space between arms widely sclerotized basally; 11–12 long colorless sensilla just posterior to basal juncture area (Fig. 5L); accessory sclerite absent. Last abdominal segment somewhat expanded ventrally forming ventral bulge, visible as small ventral papilla when viewed from lateral side. Posterior circlet with 71–73 rows of hooklets each row with up to 11 or 12 hooklets.

### Biology

**Fig. 6:**
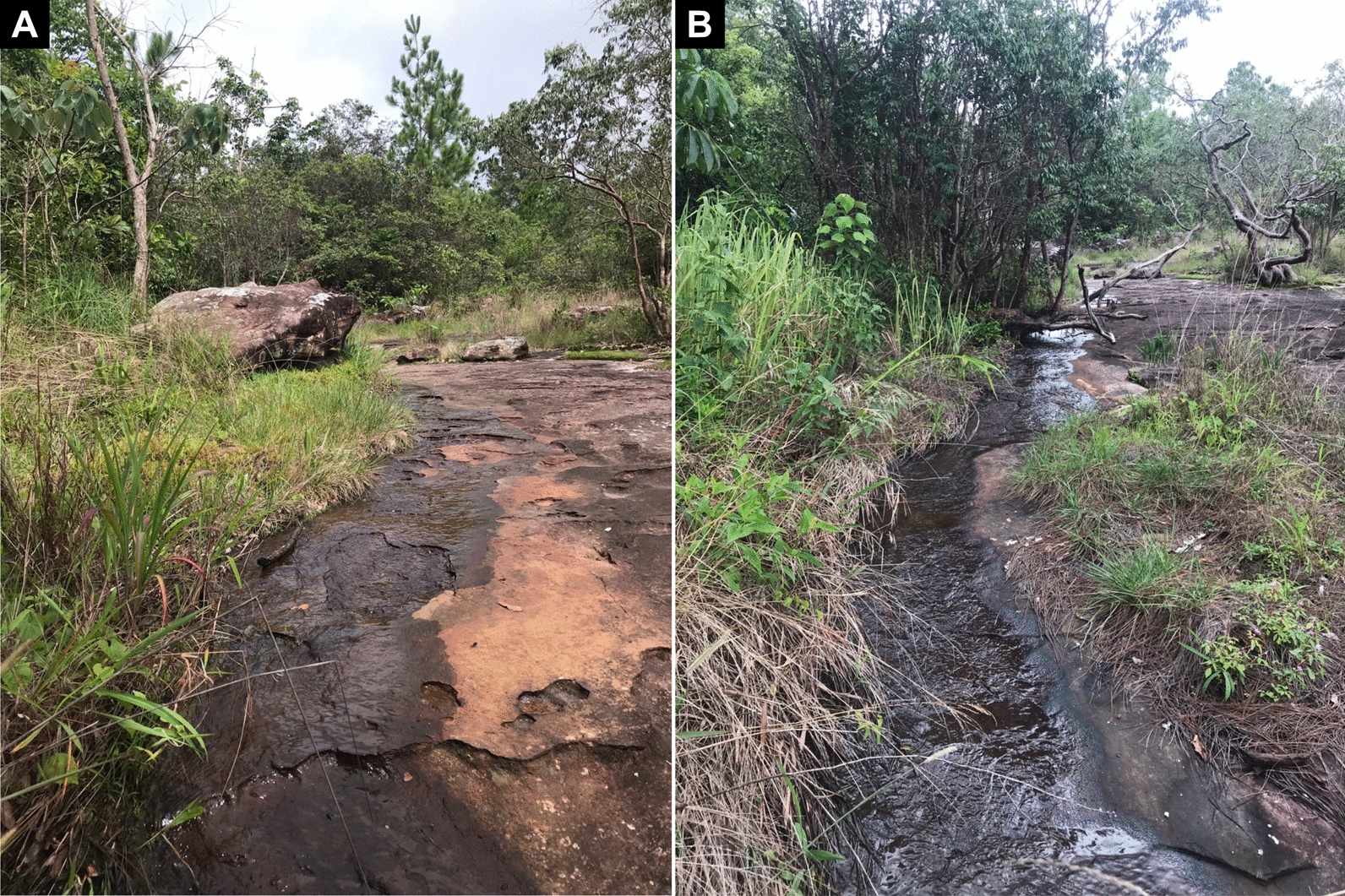
Type locality of *Simulium kittipati* sp. nov. **A** upper part and **B** lower part of the stream

The pupae and larvae of this new species were collected from fallen leaves, trailing grasses and rock surfaces in a small, moderately flowing seasonal stream (width 35 cm, depth 2 cm, bottom rocky, 18 °C, exposed to the sunlight, elevation 1123 m, 17° 00′ 27.6ʺ N 100° 59′ 21.0ʺ E) (Fig. 6). The associated species are *S*. *aureohirtum* (14 pupae and seven larvae), *S*. *feuerborni* Edwards, 1934 (complex) (six pupae and nine larvae) and *S*. *yuphae* Takaoka & Choochote, 2005 (two pupae and four larvae).

### Distribution

Nakhon Thai district, Phitsanulok province, central Thailand.

### ZooBank registration

Details of the new species have been submitted to ZooBank (www.zoobank.org). The Life Science Identifier (LSID) of the article is urn:lsid:zoobank.org:pub: 6F124CDB-6689-4CCA-B6B8-324FF400BC41. The LSID for the new species name *S*. *kittipati* is urn:lsid:zoobank.org:act:122A4843-96B4-40C2-A7EE-9E2CF9180F54.

### Etymology

The species name, *kittipati*, is in honor of Dr. Kittipat Aupalee, Department of Parasitology, Faculty of Medicine, Chiang Mai University, for his great contribution to the study of black flies in Thailand.

### Genetic distances

**Fig. 7:**
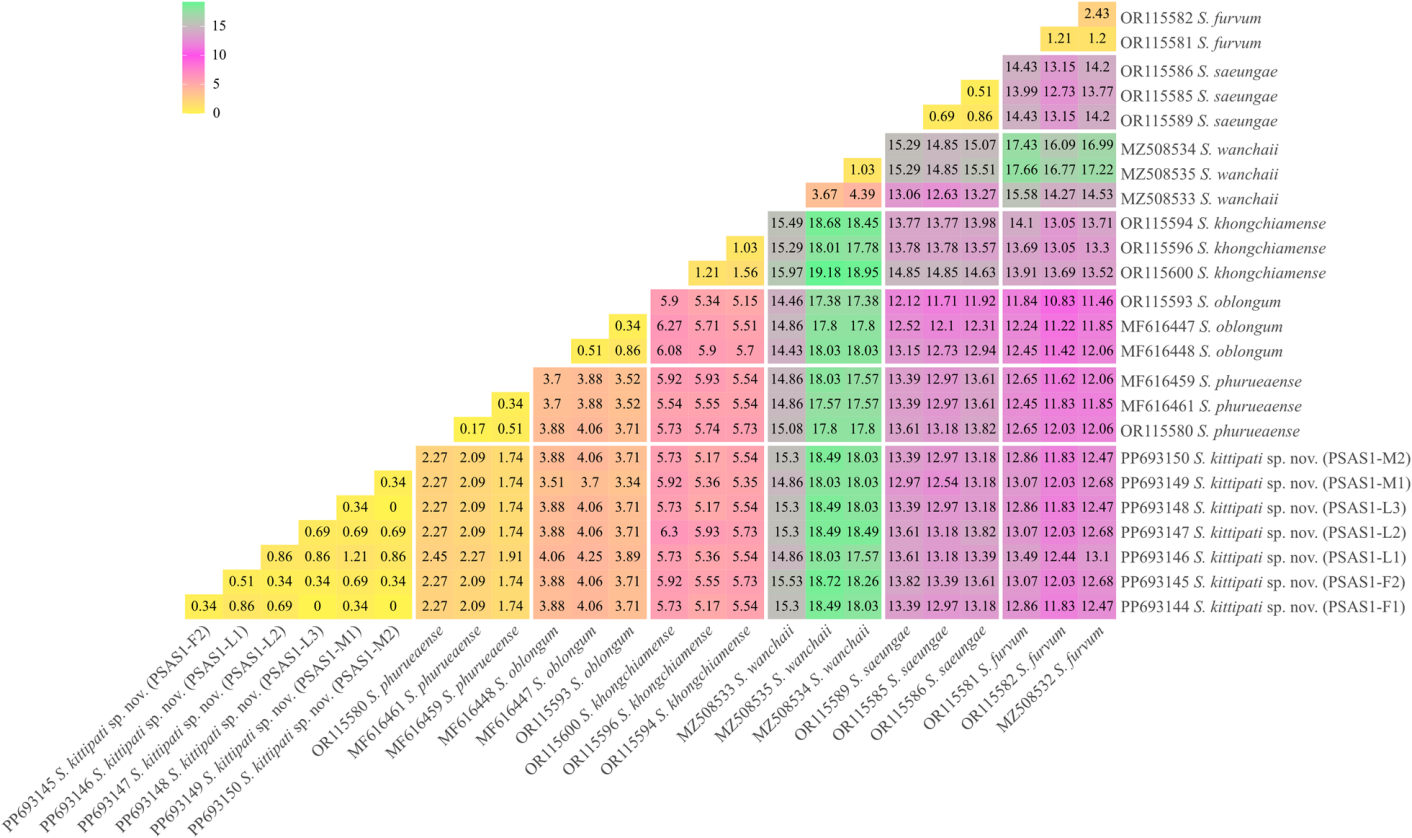
Colour heatmap illustrating the pairwise genetic distances between seven known related species members of the subgenus *Asiosimulium*. The shade of colour indicates the levels of intraspecific and interspecific genetic distances (%)

Pairwise comparisons of genetic distance within and between nominal species of the subgenus *Asiosimulium* based on the *COI* gene are shown in Fig. 7. The intraspecific genetic distance of the new species, *S*. *kittipati* sp. nov. ranged from 0.00 to 1.21% (average 0.52%), whereas the interspecific genetic distance ranged from 1.74 to 18.72% (average 9.10%). Overall, the maximum intraspecific genetic distances of all species were less than 3%, except for *S*. *wanchaii* (4.39%). Meanwhile, the interspecific genetic divergence ranged from 1.74 to 19.18%, with an average of 10.87%. A low level of interspecific genetic distance (< 3%) was observed between *S*. *kittipati* sp. nov. and *S*. *phurueaense*, indicating that they are closely related species. This result also supports the morphological description of *S*. *kittipati* sp. nov. revealing that it is similar to *S*. *phurueaense* in many characteristics. Furthermore, we found that in this subgenus, the maximum intraspecific distances exceeded the minimum interspecific distances, leading to the absence of a clear DNA barcode gap for reliable species differentiation.

### Phylogenetic analyses based on the *COI* gene

**Fig. 8:**
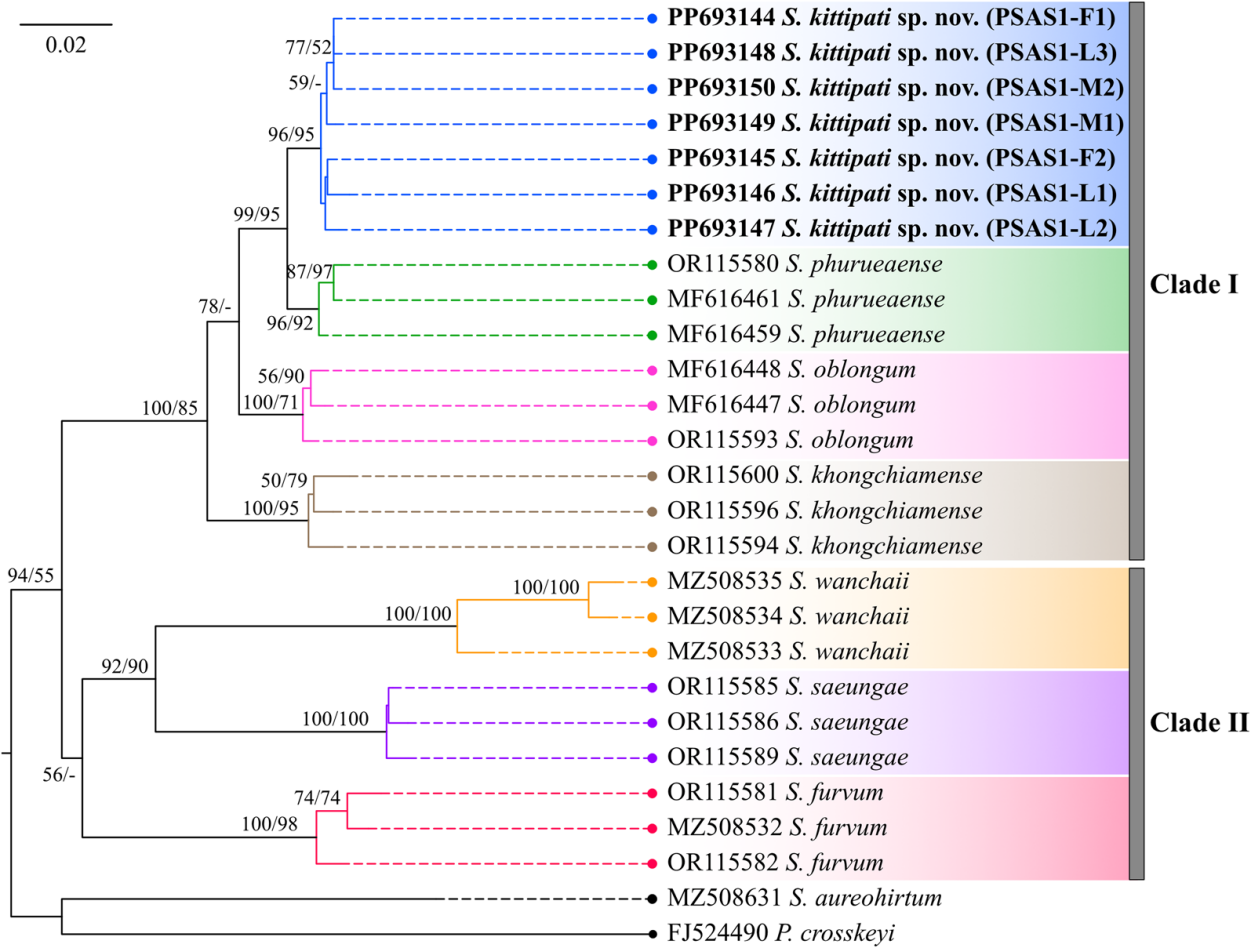
Rooted neighbour-joining phylogenetic tree based on 586 bp fragments of *COI* gene showing the genetic position of *S*. *kittipati* sp. nov. (in bold) and its six other related species within the subgenus *Asiosimulium*. Numbers near the branches represent bootstrap values (NJ/ML; ≥ 50%). The scale bar indicates 0.02 substitution of the nucleotide

Both the NJ and ML trees revealed similar topologies, delineating seven species members of the subgenus *Asiosimulium* into two main clades (Fig. 8). Four species, including *S*. *kittipati* sp. nov., *S*. *phurueaense*, *S*. *oblongum* and *S*. *khongchiamense*, formed one clade (Clade I), while the remaining three species, namely *S*. *wanchaii*, *S*. *saeungae*, and *S*. *furvum*, formed another clade (Clade II). All seven species were recovered as monophyletic with strong branch supports and could be clearly differentiated from each other despite no clear barcode gap. The newly discovered species was positioned as a sister taxon to *S*. *phurueaense*, suggesting a close genetic relationship between the two species.

This observation was in complete agreement with the morphological comparisons demonstrating a high degree of similarity between the new species and *S*. *phurueaense* across all life stages (except for the egg).

## Discussion

### Morphological analysis

*Simulium kittipati* is assigned to the subgenus *Asiosimulium*, defined by Takaoka and Choochote [18], as it possesses a combination of the following characteristics: the pleural membrane and katepisternum bare and the basal portion of the radius haired in the female and male; the cibarium with numerous spinous processes medially (Fig. 2F), claw with a large basal tooth (Fig. 2P) and spermatheca ellipsoidal (Fig. 2W) in the female; ventral plate concave medially on the posterior margin and with numerous fine short to long setae (Fig. 3O), parameral hooks absent (Fig. 3S) in the male; gill filaments of arborescent type with 28–30 filaments (Fig. 4J) in the pupa; and postgenal cleft deep (Fig. 5E and J) and ventral papillae present in the larva (Table S1).

*Simulium kittipati* sp. nov. is similar to *S*. *phurueaense* in many characteristics, including the scutum with three longitudinal vittae in females and males, female genital fork with an incision basally, male genitalia, number of pupal gill filaments (28–30 filaments in this new species vs. 30–32 filaments in *S*. *phurueaense*) and postgenal cleft deep but not reaching the posterior margin of the hypostoma and dark sheath of the subesophageal ganglion in larva [28]. However, this new species is clearly distinguished from the latter known species by several characteristics. In females, the shape of the spermatheca is ellipsoidal (Fig. 2W) in this new species, whereas it is globular in *S*. *phurueaense*. The female cercus is unusually long (Fig. 2U, V), about 1.6 times as long as its basal width (2.0 times in *S*. *phurueaense*). In males, the apical portion of the median sclerite is broad, with several sutures and upturned apically (Fig. 3R) in this new species, while in *S*. *phurueaense*, the median sclerite has no sutures and is not upturned apically. At the pupal stage, the head (Fig. 4C) and thorax are densely covered with tubercles in this new species (moderately covered with tubercles in *S*. *phurueaense*). In the larval stage, the number of rows of hooklets of the posterior circlet and the number of hooklets per row differ, with 71–73 rows and 11–12 hooklets in this new species compared with 80 rows and 15 hooklets in *S*. *phurueaense* [28].

The number of pupal gill in *S*. *kittipati* sp. nov. is similar to that of *S*. *suchitrae* (Table S1) but it differs from the latter species by the gill lacking an enlarged transparent bulbous basal fenestra basally [22]. This new species is readily distinguished from the five other related species by the number of gill filaments (31–33 in *S. oblongum* and *S*. *khongchiamense*, 42–56 in *S*. *saeungae*, 22 in *S*. *furvum* and 19 in *S*. *wanchaii*) [16–18, 23, 27].

This new species is distinguished from *S*. *shanense*, whose female, pupal and larval stages are unknown, by the number of the male upper-eye (large) facets in 20 vertical columns and 21 horizontal rows (14 vertical columns and 15 horizontal rows in *S*. *shanense*) and posterior margin of the ventral plate in ventral view moderately concave medially (slightly concave in *S*. *shanense*) [21].

### Genetic analysis

In this study, a high level of intraspecific genetic divergence (> 3%) within *S*. *wanchaii* was detected suggesting possible cryptic diversity, but further morphological and molecular studies are needed to verify this observation. Furthermore, the sequence analysis revealed the absence of a clear DNA barcode gap for reliable species differentiation of members in the subgenus *Asiosimulium*. Three main factors have been reported as contributing to the absence of this barcode gap: (1) the recent/rapid species divergence from the most recent common ancestor (MRCA), (2) the probable existence of cryptic species diversity and (3) errors in sample identification by taxonomists [37, 38]. Other factors, including incomplete lineage sorting, interspecific introgression and inadequate signal of DNA barcode being used, have also been proposed as causes of low interspecific genetic distance, which lead to no barcode gap [39]. In this study, the overlap between maximum intra- and minimum interspecific genetic distances is attributed to both the high intraspecific variations of certain species and low interspecific variations between closely related species [40]. Specifically, the low interspecific genetic distance (< 2%) observed between *S*. *kittipati* sp. nov. and *S*. *phurueaense* is most likely owing to inadequate signal of the mitochondrial *COI* gene because these species have recently diverged from a common ancestor. On the other hand, the high intraspecific genetic distance of *S*. *wanchaii* is possibly caused by the presence of an unrecognized species which are separated genetically but very similar or even identical in morphology.

Unfortunately, in this study, two valid species, *S*. *shanense* and *S*. *suchitrae*, of the subgenus *Asiosimulium* were not included in the phylogenetic analyses owing to the unavailability of their *COI* sequences in the GenBank database. Further investigations incorporating all species members of the subgenus *Asiosimulium* will shed light on the phylogenetic relationships within this subgenus.

**Identification key for species of the subgenus** ***Asiosimulium*** (updated from [17]).

**Table:** 

Adult females*	
1	Cercus very elongated, about twice as long as its basal width ………………….………… 2 Cercus normal or somewhat elongated, less than twice as long as its basal width ………… 3
2 (1)	Mandible with 20 inner and 12–13 outer teeth; highland species found at more than 1100 m above sea level ………………………………………………………………………. *S*. *phurueaense* Mandible with 22–24 inner and 8–10 outer teeth; lowland species found at less than 1000 m above sea level …………………………………………………………………………… *S*. *oblongum*
3 (1)	Abdominal segment five shiny dorsally ………………..…………………………………….. 4 Abdominal segment five dull dorsally………………………………………………………….. 6
4 (3)	Spermatheca ellipsoidal ………………….………………………………. *S*. *kittipati*** sp. nov** Spermatheca globular …………………………………………..…………..………………. 5
5 (4)	Cercus normal, 1.2 times as long as its basal width; genital folk without incision between arm ……………………………………………………………………………………………. *S*. *wanchaii* Cercus elongated, 1.9 times as long as its basal width; genital folk with deep incision between arm ………………………………………………………………………………….*S*. *khongchiamense*
6 (3)	Spermatheca globular …………………………………………………………….…………… *S*. *saeungae* Spermatheca pear-shaped ……………………………………………………..……….…….. 7
7 (6)	Arm of genital folk with stout projection ……………………………………………………….. *S*. *furvum* Arm of genital folk without projection ………………………………………………………..*S*. *suchitrae*
	*The female of *S*. *shanense* is unknown
**Adult males**^†^	
1	Hind basitarsus slightly widened, 0.80 times as wide as hind tibia ……….…………….…. 2 Hind basitarsus much widened, 0.90–1.00 times as wide as hind tibia ………………..…… 5
2 (1)	Sensory vesicle 0.35 times as long as third palp segment ….………………..* S*. * phurueaense* Sensory vesicle shorter than 0.30 times of third palp segment ……………………………………… 3
3 (2)	Abdominal segments five to eight without pair of shiny dorsolateral patches *…………… S*. *oblongum* Abdominal segments five to eight each with pair of shiny dorsolateral patches …………………………. 4
4 (3)	Upper-eye (large) facets in 20 vertical columns, median sclerite upturned apically ……… …………………………………………………………………………… *S*. *kittipati*** sp. nov** Upper-eye (large) facets in 17–18 vertical columns, median sclerite normally *………………* ……………………………………………………………………………………..*S*. *khongchiamense*
5 (1)	Abdominal segments five to eight each with pair of shiny dorsolateral patches ……..……………….. 6 Abdominal segments five to eight without pair of shiny patches …………………………..………………. 7
6 (5)	Abdominal segments three and four with pair of shiny dorsolateral patches ………………….. *S*. *shanense* Abdominal segments three and four without pair of shiny dorsolateral patches ………………* S*. *saeungae*
7 (5)	Upper-eye (large) facets in 18 or 19 vertical columns …………………………………… *S*. *wanchaii* Upper-eye (large) facets in 16 vertical columns ……………………………………………… *S*. *furvum*
	^†^The male of *S*. *suchitrae* is unknown
**Pupae**^‡^	
1	Head integument without tubercles ……………………………………….………………………… 2 Head integument with tubercles ……………………………………….………………………………… 3
2 (1)	Gill with 28 filaments; basal fenestra at base of gill extremely bulbous ………… *S*. *suchitrae* Gill with 42–46 filaments; basal fenestra at base of gill normal, small…………… *S*. *saeungae*
3 (1)	Thoracic integument sparsely covered with tubercles …………………………*S*. *khongchiamense* Thoracic integument moderately or densely covered with tubercles ……………….…… 4
4 (3)	Thoracic integument densely covered with tubercles …………………… *S*. *kittipati*** sp. nov** Thoracic integument moderately covered with tubercles …………………………………… 5
5 (4)	Gill with 31–33 filaments …………………………..………. *S*. *oblongum* and *S*. *phurueaense* Gill with 19 or 22 filaments ……………………………….………….……………………. 6
6 (5)	Gill with 19 filaments ………………………………..…..…………….….………… *S*. *wanchaii* Gill with 22 filaments ……………………….……………………………………… *S*. *furvum*
	^‡^The pupa of *S*. *shanense* is unknown
**Mature larvae**^§^	
1	Histoblast of pupal gill with basal fenestra extremely bulbous ………………………. *S*. *suchitrae* Histoblast of pupal gill with basal fenestra normal, small …………………………………………… 2
2 (1)	Postgenal cleft with pigmented sheath of subesophageal ganglion ………………………………. 3 Postgenal cleft without pigmented sheath of subesophageal ganglion ……….….………… 6
3 (2)	Histoblast of pupal gill with 22 filaments ………………………….……………… *S*. *furvum* Histoblast of pupal gill with 28–33 filaments ………………………….……………..…… 4
4 (3)	Histoblast of pupal gill with 28–30 filaments ……………..…….……… *S*. *kittipati*** sp. nov** Histoblast of pupal gill with 31–33 filaments ………………………………..………..…… 5
5 (4)	Labral fan with 42–45 main rays ………………………………………………………….. *S*. *phurueaense* Labral fan with 37–39 main rays ……………………………………………………..*S*. *khongchiamense*
6 (2)	Labral fan with 30–33 primary rays ………………………………………….… *S*. *saeungae* Labral fan with 38–45 primary rays …………………………………….…………………. 7
7 (6)	Labral fan with 38–40 primary rays; mandible with supernumerary serrations … *S*. *wanchaii* Labral fan with 43–45 primary rays; mandible without supernumerary serration ………….. ………………………………………………………………………………….. *S*. *oblongum*
	^§^The larva of *S*. *shanense* is unknown

## Conclusions

*Simulium kittipati* sp. nov. was described as the ninth species of the subgenus *Asiosimulium*. The distinct species status of this new species was supported by both morphological observation and genetic investigation on the basis of the *COI* gene.

## Supplementary Information

Supplementary Material 1. Table S1 Taxonomic characters of nine closely related species in the subgenus *Asiosimulium*.

## Data Availability

The authors confirm that the data supporting the findings of this study are available within the article. All sequences obtained from the study were deposited in the GenBank database under the accession numbers PP693144–PP693150.
