# Supplementary material for: Morphological description and genetic analysis of a new black fly species (Diptera: Simuliidae) in the subgenus Asiosimulium from central Thailand
Source: Parasit Vectors. 2024 Sep 5;17:379. doi: 10.1186/s13071-024-06441-z (PMC11378507; doi:10.1186/s13071-024-06441-z)
Supplement: Supplementary file 1 — Supplementary Material 1. Table S1 Taxonomic characters of nine closely related species in the subgenus Asiosimulium. [file 13071_2024_6441_MOESM1_ESM.docx]

**Table S1** Taxonomic characters of nine closely related species in the subgenus *Asiosimulium*

| **Life stages** | ***S. kittipati* sp. nov.** | ***S. phurueaense*** | ***S. oblongum*** | ***S. khongchiamense*** | ***S. furvum*** | ***S. wanchaii*** | ***S. saeungae*** | ***S. shanense*^1^** | ***S. suchitrae*^2^** |
| --- | --- | --- | --- | --- | --- | --- | --- | --- | --- |
| **Female** |  |  |  |  |  |  |  |  |  |
| Length of sensory vesicle vs. length of third palpomere | 0.25–0.35 times | 0.32 times | 0.36 times | 0.38–0.46 times | 0.24 times | 0.31 times | 0.25–0.29 times | - | 0.3 times |
| No. of spinous processes in cibarium | 116–119 | 92–115 | 100–120 | 59–94 | 111 | 96–102 | 64–94 | - | 69 |
| Incision between arms  of genital fork | Present | Present | Present | Present | Absent | Absent | Absent | - | Absent |
| Spermatheca | Ellipsoidal | Globular | Globular | Globular | Pear shape | Globular | Globular | - | Pear shape |
| **Male** |  |  |  |  |  |  |  |  |  |
| Number of upper-eye facets;  Vertical column/ Horizontal row | 20 V.  21 H. | 19 V.  20 H. | 19 V.  19 H. | 17–18 V.  18–19 H. | 16 V.  17 H. | 18–19 V.  19–20 H. | 16–17 V.  17–19 H. | 14 V.  15 H. | - |
| Scutum pattern | 3 longitudinal vittae | 3 longitudinal vittae | 3 longitudinal vittae | 3 longitudinal vittae | Absent | 2 longitudinal vittae | 3 longitudinal vittae | 2 longitudinal vittae | - |
| Median sclerite | Upturned apically | Normal | Normal | Normal | Normal | Normal | Normal | Normal | - |

| **Life stages** | ***S. kittipati* sp. nov.** | ***S. phurueaense*** | ***S. oblongum*** | ***S. khongchiamense*** | ***S. furvum*** | ***S. wanchaii*** | ***S. saeungae*** | ***S. shanense*^1^** | ***S. suchitrae*^2^** |
| --- | --- | --- | --- | --- | --- | --- | --- | --- | --- |
| **Pupae** |  |  |  |  |  |  |  |  |  |
| No. of gill filaments | 28–30 | 30–32 | 31–33 | 32–33 | 22 | 18 | 42–56 | - | 28 |
| Tubercle on head and thoracic integuments | Densely | Moderately | Moderately | Sparsely | Moderately | Moderately | Bare | - | Bare |
| **Larvae** |  |  |  |  |  |  |  |  |  |
| No. of primary rays of labral fan | 41–42 | 42–45 | 43–45 | 37–39 | 45–49 | 38–40 | 30–33 | - | 30 |
| Postgenal cleft: Not reaching or reaching posterior margin of hypostoma | Not | Not | Reaching | Reaching | Reaching | Reaching | Reaching | - | Reaching |
| Pigmented on subesophageal ganglion | Dark | Dark | Absent | Dark | Dark | Absent | Absent | - | Absent |
| No. of rows of hooklets and no. of hooklets per row of posterior circlet | 71–73 rows  11–12 hooklets | 80 rows  15 hooklets | 76 rows  15 hooklets | 72–77 rows  12–13 hooklets | 80 rows  15 hooklets | 80–81 rows  14–16 hooklets | 83 rows  15–16 hooklets | - | 90 rows  16 hooklets |

**Table S1** (continued)
